# Supplementary material for: CA-IX-Expressing Small Extracellular Vesicles (sEVs) Are Released by Melanoma Cells under Hypoxia and in the Blood of Advanced Melanoma Patients
Source: Int J Mol Sci. 2023 Mar 24;24(7):6122. doi: 10.3390/ijms24076122 (PMC10094632; doi:10.3390/ijms24076122)
Supplement: Supplementary file 1 [file ijms-24-06122-s001.zip › ijms-2213281-supplementary.pdf]

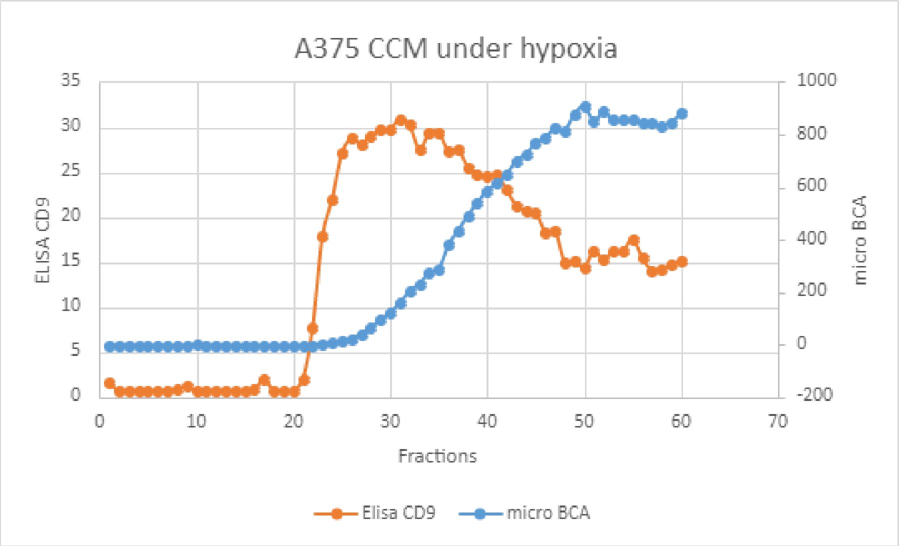
A375 CCM under hypoxia
ELISA CD9
micro BCA
Fractions
Elisa CD9
micro BCA

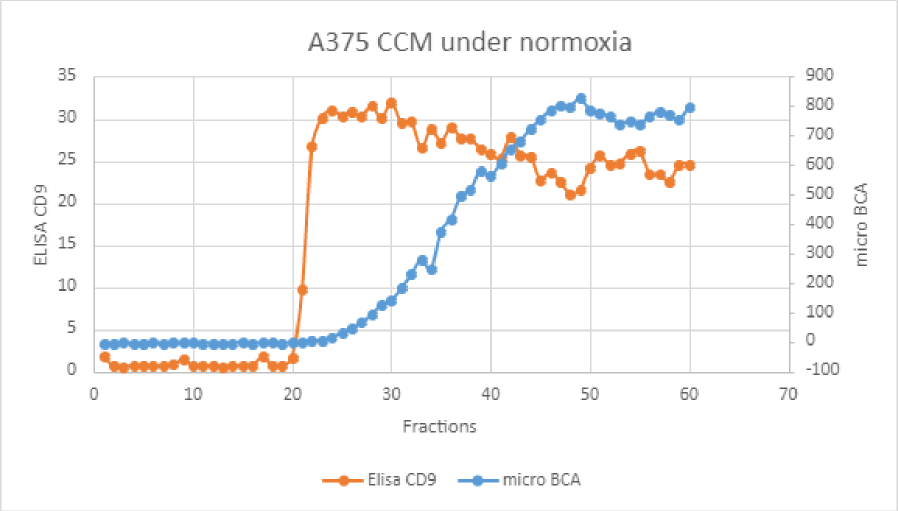
A375 CCM under normoxia
ELISA CD9
micro BCA
Fractions
Elisa CD9
micro BCA

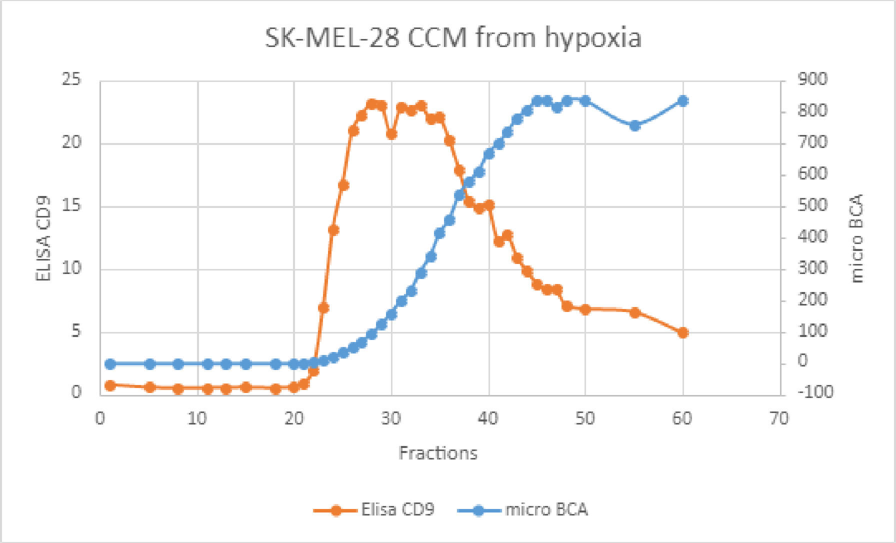
SK-MEL-28 CCM from hypoxia
ELISA CD9
micro BCA
Fractions
Elisa CD9
micro BCA

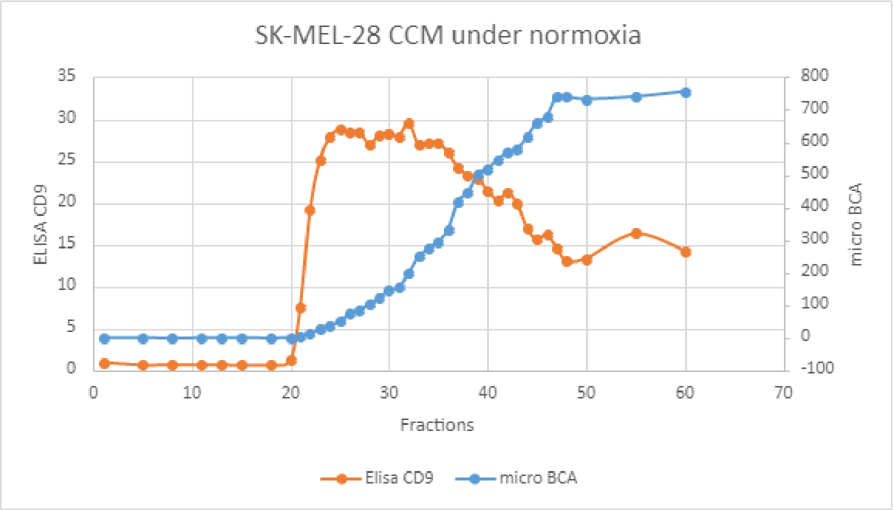

Figure S1. SEC chromatography elution profiles of CCM samples from melanoma cells, under normoxia and hypoxia.

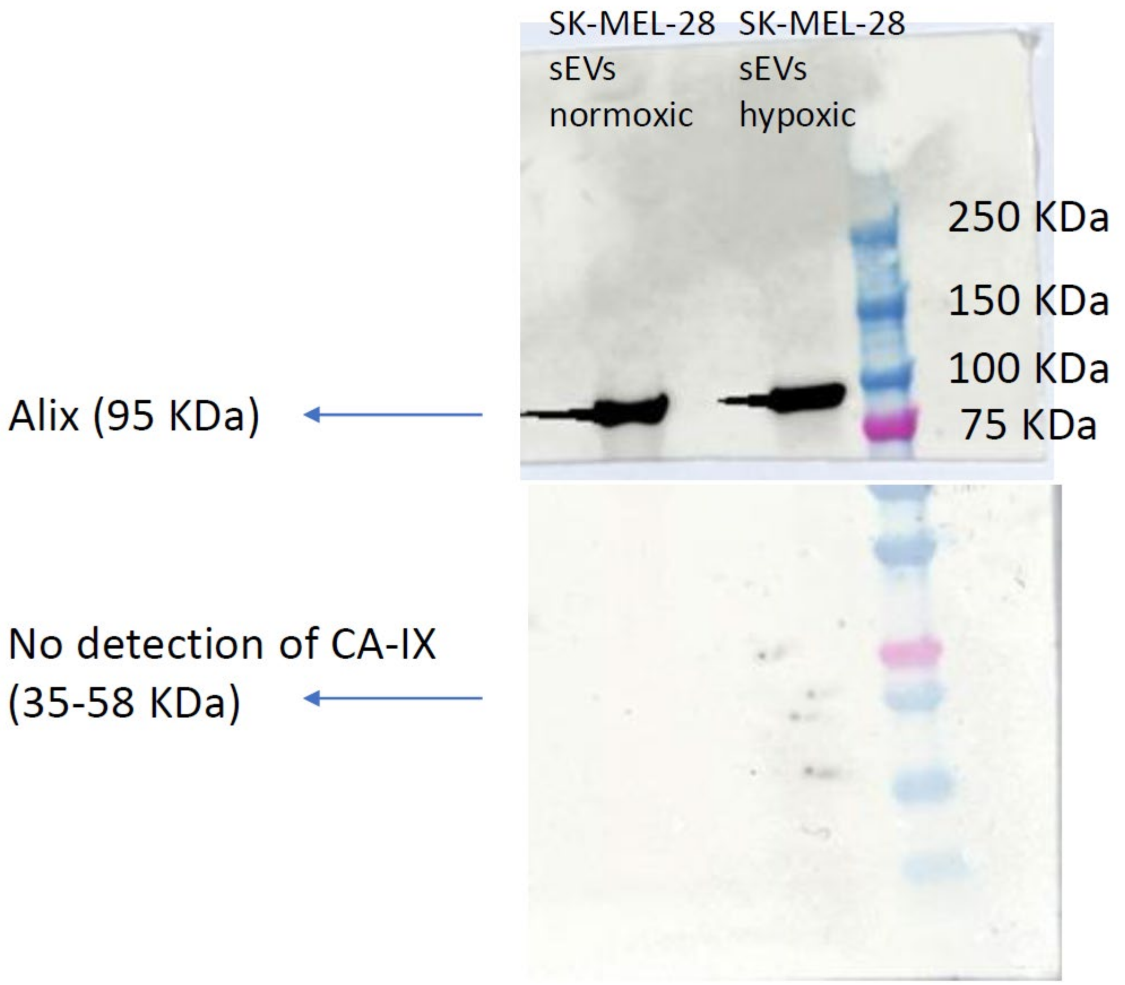

Figure S2. The CA-IX protein was not found on sEVs from the hypoxic SK-MEL-28 cells, while Alix was expressed in both conditions.
